# *Escherichia coli* adhesion protein FimH exacerbates colitis via CD11b^+^CD103^-^ dendritic cell activation

**DOI:** 10.3389/fimmu.2023.1284770

**Published:** 2023-11-22

**Authors:** Wei Zhang, Eun-Koung An, So-Jung Kim, Hae-Bin Park, Peter C. W. Lee, Jun-O Jin

**Affiliations:** ^1^ Shanghai Public Health Clinical Center, Shanghai Medical College, Fudan University, Shanghai, China; ^2^ Department of Microbiology, ASAN Medical Center, University of Ulsan College of Medicine, Seoul, Republic of Korea; ^3^ Department of Biochemistry and Molecular Biology, ASAN Medical Center, University of Ulsan College of Medicine, Seoul, Republic of Korea

**Keywords:** colitis, dendritic cell, FimH, Th1, Th17

## Abstract

**Introduction:**

Immune stimulators are used to improve vaccine efficiency; however, they are accompanied by various side effects. In previous studies, we reported that the *Escherichia coli* adhesion protein, FimH, induces immune activity; however, we did not examine any side effects in colon inflammation.

**Methods:**

FimH was administered orally or intraperitoneally (*i.p.*) to mice with dextran sulfate sodium (DSS)-induced colitis, and changes in symptoms were observed. Immune cells infiltrated into the colon after the induction of colon inflammation were analyzed using a flow cytometer. Changes in Th1 and Th17 cells that induce colitis were analyzed. Further, mesenteric lymph node (mLN) dendritic cells (DCs) activated by FimH were identified and isolated to examine their ability to induce T-cell immunity.

**Results:**

FimH oral and *i.p.* administration in C57BL/6 mice did not induce inflammation in the colon; however, DSS-induced colitis was exacerbated by oral and *i.p.* FimH administration. FimH treatment increased immune cell infiltration in the colon compared to that in DSS colitis. Th1 and Th17 cells, which are directly related to colitis, were increased in the colon by FimH; however, FimH did not directly affect the differentiation of these T cells. FimH upregulated the CD11b^+^CD103^-^ DC activity in the mLNs, which produced the signature cytokines required for Th1 and Th17. In addition, isolated CD11b^+^CD103^-^ DCs, after stimulation with FimH, directly induced Th1 and Th17 differentiation in a co-culture of CD4 T cells.

**Conclusion:**

This study demonstrated the side effects of FimH and indicated that the use of FimH can aggravate the disease in patients with colitis.

## 1 Introduction

Immune stimulators are used to improve the efficiency of vaccines and treat cancer (1, 2). They are essential to induce specific immune activity against non-immunogenic antigens, such as cancer antigens (3). Immune stimulators mostly target innate immune cells, such as macrophages and dendritic cells (DCs), to induce immune activation (4, 5). Most ligands that can stimulate pattern recognition receptors (PRRs) in innate immune cells have been developed as immune stimulators (2). These immune stimulators positively induce immune activation against antigens; however, they can cause side effects such as inflammation or autoimmune diseases (6–9).

Toll-like receptors (TLRs) are well-studied PRRs, and various ligands have been identified (10). Imiquimod is a TLR7/8 ligand actively used in research for developing a viral vaccine; however, it induces psoriasis, a skin inflammatory disease (8). Unmethylated single-stranded CpG in bacterial DNA is used for immunotherapy and vaccines by stimulating TLR9; however, it is known to induce inflammation (9). Lipopolysaccharide (LPS), a ligand of TLR4, is an endotoxin as its immune activation ability is strong enough to induce a cytokine storm or sepsis (11). Recently, we showed that FimH, a protein form of the TLR4 stimulator, can be used as an adjuvant in cancer vaccines (12–14). However, the side effects of FimH, especially in inflammatory diseases, have not yet been elucidated.

Inflammatory bowel disease (IBD) is an autoimmune disease associated with inflammation of digestive tract tissues (15). Two types of IBD are available: ulcerative colitis and Crohn’s disease (15). Colitis causes inflammation in the large intestine and rectum. In contrast, Crohn’s disease can cause digestive tract inflammation, especially in the small intestine and upper gastrointestinal tract (16). IBD is an inflammatory disease that involves immune cell activation. The activity of helper T 1 cells (Th1) and Th17 cells has a direct effect on IBD (17, 18). In addition, the absence of regulatory T cells (Treg) is directly related to the occurrence of IBD (19). Many causes of IBD exist, such as drug administration and bacterial infection (15). Numerous drugs that aggravate IBD have been studied (7, 20).

DCs are specialized antigen-presenting cells involved in activating and suppressing immunity via phagocytosis and antigen presentation (21). DCs appear in different subtypes in the body, depending on the tissue in which they reside. DCs involved in immune activation are observed in secondary lymphoid organs. In contrast, immunosuppressive and immunostimulatory DCs are observed in peripheral tissues, particularly the lungs and intestines (21, 22). The three main subtypes of conventional DCs (cDCs) in the murine intestine can be divided according to their surface marker expression. Intestinal cDC1 is defined as CD103^+^CD11b^–^ cells in CD11c^+^ cells and cDC2 as CD103^–^CD11b^+^ cells (23). Gut-specific cDC2 subsets express both CD103 and CD11b (23). These DC subsets are involved in immune activation and suppression (23). Among these DCs, CD103^+^CD11b^+^ and CD103^+^CD11b^–^ DCs promote immunity suppression by inducing regulatory T cell differentiation in mesenteric lymph nodes (mLNs) (23). However, CD103^+^CD11b^+^ and CD103^+^CD11b^–^ DCs induce colitis by inducing Th17 and Th1 differentiation, respectively (24). CD103^–^CD11b^+^ DC are involved in immune activation, and in particular, they induce IBD via inducing Th1 and Th17 differentiation (24–26).

Adherent-invasive *E. coli* (AIEC) is associated with IBD (27). AIEC attaches to the intestinal epithelia, penetrates the intestinal wall, and induces inflammation (27, 28). The pilus and flagella in AIEC are the main adhesion portions for attachment to the intestinal epithelia (29). Flagellin, a flagella protein, is a well-known substance that stimulates TLR5 (30). Flagellin is known to induce immune activity; however, it is closely associated with IBD (31). Antibodies against flagellin are increased in patients with IBD, and flagellin aggravates colitis in a dextran sulfate sodium (DSS)-induced murine colitis model (32, 33). FimH, a protein involved in adhesion that is observed at the end of type I pili of AIEC (34), can induce immune activation by stimulating TLR4 and preventing colitis by binding to the intestinal epithelium and inhibiting AIEC invasion (34, 35). However, studies on the effect of FimH in the presence and exacerbation of colitis are lacking. Thus, we conducted this study under the hypothesis that FimH may aggravate colitis based on its ability to induce immune activation.

## 2 Materials and methods

### 2.1 Mice

C57BL/6 mice were purchased from the Shanghai Public Health Clinical Center (SPHCC) and Hyochang Science (Daegu, Korea). The mice were kept under pathogen-free conditions, housed in a room at 20–22°C and 50–60% humidity, and fed standard rodent chow and water. The animal experiments were approved by the Ethics Committee of SPHCC (mouse protocol number: 2021-A070-01) and the Asan Medical Center (authorization no. 2023-04-041).

### 2.2 Recombinant FimH protein purification

The recombinant FimH protein was prepared from *E. coli* (strain K21) as previously described (12). Briefly, the pET28a-FimH plasmids were constructed and transformed into BL21-competent cells. After incubation in lysogenia broth (LB) medium containing IPTG to induce expression, competent cells were harvested and disrupted using ultrasonication. The target FimH protein was purified from the cell supernatant using an Ni column and then renatured in a dialysis bag. The endotoxin contaminating the FimH protein was removed using endotoxin removal resin (Thermo Fisher Scientific, Waltham, MA, USA) according to the manufacturer’s instructions. Finally, the FimH protein was lyophilized and stored at −80°C until use.

### 2.3 Reagents and antibodies

DSS (40 kDa) was obtained from Sigma-Aldrich (St. Louis, Missouri, US). Murine monoclonal antibodies anti-IgG1 (RMG1-1), anti-IgG2a (RMG2a-62), anti-IgG2b (RMG2b-1), anti-TCR-β (H57-597), anti-TCR-γδ (GL3), anti-NK1.1 (S17016D), anti-Ly-6G (S19018G), anti-CD4 (GK1.5), anti-CD8 (53-6.7), anti-CD11c (N418), anti-CD45 (QA17A26), anti-CD11b (M1/70), anti-CD103 (W19396D), anti-CD40 (3/23), anti-CD80 (16-10A1), anti-CD86 (GL-1), anti-major histocompatibility complex (MHC) class I (AF6-88.5.3), anti-MHC class II (M5/114.15.2), anti-interferon (IFN)-γ (XMG1.2), anti-interleukin (IL)-4, IL-17, and anti-TNF-α (MP6-XT22), anti-p38 (Poly6224), anti-p38 phospho (A16016A), anti-ERK1/2 (W15133B) and anti-ERK1/2 phospho (6B8B69) were purchased from BioLegend (San Diego, CA, US). Anti-JNK (1E5) and anti-JNK phosphor (SAPKT183Y185-A11) were purchased from abcam (Cambridge, UK). Recombinant murine cytokines, IL-1β, IL-6, IL-12, IL-23, and TGF-β, were obtained from Thermo Fisher Scientific (Waltham, MA, US).

### 2.4 DSS mild colitis model

The mice were randomly divided into six groups (three mice in each group repeated the experiment twice; n = 6). The 1^st^ group was the control group, which received normal water daily. In the 2^nd^ and 3^rd^ groups, in addition to providing normal water daily, FimH (10 mg/kg) was intraperitoneally (*i.p.*) or orally administered daily. The 4^th^, 5^th^, and 6^th^ groups were administered 1.5% DSS in water for nine days to induce mild colitis. The 5^th^ and 6^th^ groups were additionally treated with FimH (10 mg/kg) *i.p.* or orally. The disease activity index (DAI) was calculated on day 9 after DSS administration, as previously described (36). The DAI was scored on a scale of 0–4 using the following parameters: loss of body weight (0, normal; 1, 0–5%; 2, 5–10%; 3, 10–20%; 4, > 20%), stool consistency (0, normal; 2, loose stools; 4, watery diarrhea), and the occurrence of gross blood in the stool (0, negative; 4, positive).

### 2.5 Hematoxylin and eosin staining

The colons were harvested and fixed with 4% paraformaldehyde. After dehydration with 100% ethanol, the tissues were embedded in paraffin and sectioned into 5 μm thickness. The sectioned colon was attached to a glass slide and deparaffinized with xylene. The sections were then stained with H&E after rehydration and examined for tissue damage. The histological scores were evaluated as previously described (37).

### 2.6 Single- cell suspension from the colon and mLN

C57BL/6 mice were orally treated with PBS, FimH (10 mg/kg), 1.5% DSS water, and FimH + 1.5% DSS water. The mice were euthanized for the T cell experiment, and the colon and mLN were harvested 3 or 5 days after consuming DSS water. DC activation was measured 24 h after the consumption of DSS water. The harvested colon, or mLN, was prepared as a single suspension by incubation with digestion buffer (DNase I and collagenase IV; both from Sigma-Aldrich) for 20 min. The remaining fat was removed with a 100 nm nylon filter, and cells were resuspended in 5 mL of 1.077 Histopaque (Sigma-Aldrich). The cell suspension was then slowly added to the upper layer of 5 mL of fresh 1.077 Histopaque. The cells were centrifuged at 1836 ×*g* for 10 min, and the cells in the supernatant at 1.077 µg/mL were harvested as leukocytes.

### 2.7 Flow cytometry analysis

The cells were pre-incubated with Fc-blocking antibodies (BioLegend) for 15 min and then stained with fluorescence-labeled antibodies at 4°C for 15 min. The stained cells were washed with PBS and resuspended in 4’,6-diamidino-2-phenylindole (DAPI) (Sigma-Aldrich) containing PBS. The cells were analyzed using a FACS LSR II (Becton Dickinson, Franklin Lakes, NJ, USA) or NovoExpress (ACEA Biosciences Inc., San Diego, CA, USA).

### 2.8 T cell differentiation

Naïve CD4 T cells were isolated from spleen and LN using naïve CD4 T cell isolation kits (Miltenyi Biotec). CD4 T cells (1 × 10^6^) were incubated in a 5 μg/mL anti-CD3 Ab and anti-CD28 Ab- coated 24- well plate for three days. To induce Th1 cell differentiation, the cells were treated with 50 ng/mL IL-12. Th17 cells were differentiated using a combination of IL-1β (10 ng/mL), IL-6 (10 ng/mL), IL-23 (20 ng/mL), and TGF-β (20 ng/mL). Three days after incubation, the cells were analyzed for intracellular cytokine production.

### 2.9 Intracellular T cell staining

To analyze intracellular cytokine-producing T cells, mLN cells were stimulated with 50 ng/mL phorbol 12-myristate 13-acetate (PMA; Sigma-Aldrich) and 1 mM ionomycin (Sigma-Aldrich) for 4 h. The cells were treated with 2 μM monensin (BioLegend), a Golgi stop for 2 h before harvesting. Cells were stained with anti-CD3, anti-CD4, anti-CD8, and anti-TCR-β antibodies. The cells were washed with PBS, and free antibodies were removed. The cell pellet was fixed with fixation buffer (BioLegend) for 20 min. After washing with 500 μL of permeabilization buffer (BioLegend), the cells were stained with intracellular antibodies in Perm/Wash buffer and T cells with PE-Cy7-anti-IFN-γ, PerCp5.5-anti-IL-4, and PE-anti-IL-17A for 30 min. Dead cells were isolated using the Zombie Violet Fixable Viability Kit (BioLegend). The cells were analyzed using a NovoCyte Flow Cytometer and NovoExpress Software (ACEA Biosciences).

### 2.10 Real-time PCR

Total RNA was isolated from bone marrow derived dendritic cell (BMDC), and cDNA was synthesized using Oligo (dT) and M-MLV reverse transcriptase (Promega, Madison, Wisconsin, US). cDNA was annealed and extended at 60°C on a LightCycler 480 Real-Time PCR System (Roche, Basel, Switzerland). Primer sequences are provided in **Table S1**.

### 2.11 Enzyme-linked immunosorbent assay

The concentration of cytokines was measured using the culture medium of cells or serum from mice. The sample was measured in triplicate, and the concentration was measured according to the experimental method provided by the manufacturer (BioLegend).

### 2.12 mLN DC analysis

mLN cells were stained with anti-CD11b, anti-CD11c, anti-CD45, and anti-CD103 antibodies. The CD11c^+^CD45^+^ cells were defined as mLN DCs, and the cells further divided CD11b^+^CD103^-^, CD11b^+^CD103^+^, and CD11b^-^CD103^+^ cells as subsets of DCs. In the subsets of mLN DCs, co-stimulatory and MHC molecule expression were analyzed via flow cytometry.

### 2.13 mLN DC and CD4 T cell co-culture

The stimulated mLN DC subsets were sorted using a cell sorter (FACS Aria II). The isolated mLN subsets (1 × 10^4^) were co-cultured with purified CD4 T cells (2.5 × 10^5^) in 96- well plates. Three days after incubation, the cells were harvested and stained for intracellular cytokine production using a NovoCyte Flow Cytometer and NovoExpress Software (ACEA Biosciences).

### 2.14 Statistical analysis

All data are expressed as the mean ± standard error of the mean (SEM). One- or two-way ANOVA (Tukey multiple comparison test) and the Mann–Whitney *t*-test were used to analyze the data sets. Statistical significance was set at *p* < 0.05.

## 3 Results

### 3.1 FimH exacerbated DDS-induced colitis in mice

**Figure 1:**
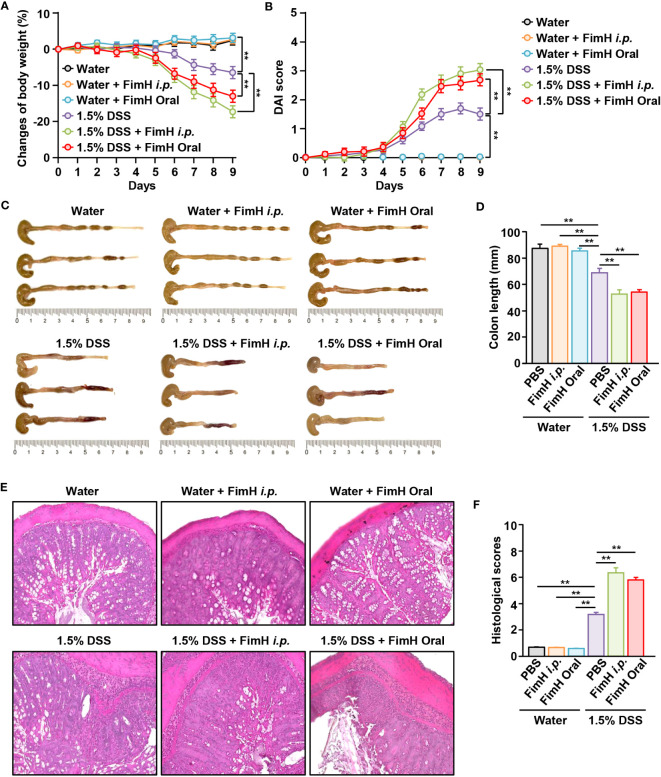
FimH administration exacerbates dextran sulfate sodium (DSS)-induced colitis in mice. C57BL/6 mice were administered tap water or 1.5% DSS-containing water for nine days. The mice were additionally administered FimH orally or intraperitoneally (*i.p*.) daily (10 mg/kg). **(A)** Body weight changes in the indicated groups (n = 6). **(B)** The disease activity index (DAI) was scored. **(C)** Photographs of the colon. **(D)** Length of the colon was measured on day nine after DSS and FimH administration. **(E)** Hematoxylin and eosin (H&E) staining images of colon sections (80 × magnification). **(F)** Histological scores were measured. Data represent the average of the analyses of two independent experiments (n = 6). Data shown are the mean ± standard error of the mean (SEM). ** p < 0.01.

To confirm the possibility of FimH-induced intestinal inflammation, normal colitis in the presence of FimH was compared with DSS-induced colitis. No change in body weight was observed in mice administered oral or intraperitoneal FimH daily (**Figure 1A**). However, weight loss in mice fed drinking water containing 1.5% DSS was more severely reduced by oral and *i.p.* administration of FimH (**Figure 1A**). In addition, the DAI score was considerably increased by the administration of FimH compared to that in DSS alone (**Figure 1B**). Consistent with changes in body weight, oral or *i.p.* administration of FimH to control mice did not promote any decrease in colon length (**Figures 1C, D**). In mice administered 1.5% DSS water, the length of the colon was reduced compared to that in the control mice (**Figures 1C, D**). In addition, oral or *i.p.* FimH administration further decreased colon length compared to 1.5% DSS water mice (**Figures 1C, D**). H&E staining of the colon tissue showed inflammation in mice that consumed 1.5% DSS water, which was aggravated by oral or *i.p.* FimH treatment (**Figures 1E, F**). Thus, these data indicate that FimH aggravates colitis in the presence of inflammation in the colon.

### 3.2 FimH elicited additional immune cell infiltration in the colon of DSS-colitis mice

**Figure 2:**
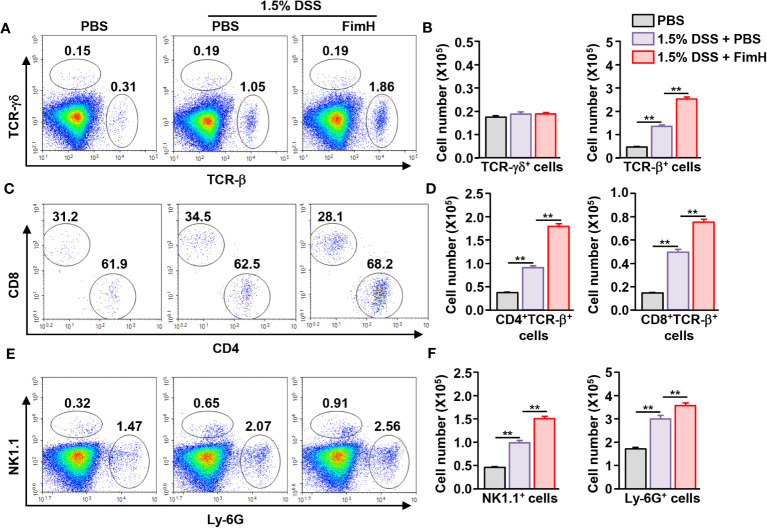
FimH promoted immune cell infiltration in the colon. **(A)** Frequency and population of colon- infiltrated TCR-γδ^+^ and TCR-β^+^ cells. **(B)** Absolute numbers of TCR-γδ^+^ and TCR-β^+^ cells in the colon. **(C)** CD4^+^ and CD8^+^ cells in TCR-β^+^ cells were shown. **(D)** Mean number of CD4^+^ and CD8^+^ cells in TCR-β^+^ cells. **(E)** Colon-infiltrated NK1.1^+^ and Ly-6G^+^ cells were analyzed using flow cytometry. **(F)** Absolute numbers of NK1.1^+^ and Ly-6G^+^ cells in the colon. Data are the average of two independent experiments (n = 6) analyses. ** p < 0.01.

Next, we examined immune cell infiltration in the colon. Colonic infiltration numbers and frequencies of TCR-β-expressing T cells were markedly increased in mice that consumed DSS water compared to those in mice that consumed normal water (**Figures 2A, B**). Single-cell suspension of colon cells was analyzed on a flow cytometer to determine the number of infiltrated immune cells (**Figure S1A**). Total cell numbers in the colon were not considerably changed by FimH administration compared to that of 1.5% DSS water control (**Figure S1B**). Oral FimH administration resulted in more TCR-β cell infiltration in the colon than in mice that consumed 1.5% DSS water (**Figures 2A, B**). In addition, CD4 and CD8 T cells were increased by FimH in DSS water mice (**Figures 2C, D**). Furthermore, DSS water intake induced NK1.1^+^ and Ly-6G^+^ cell infiltration in the colon, and the infiltration of these cells was elevated by FimH (**Figures 2E, F**). Therefore, these data suggest that FimH further enhanced the infiltration of immune cells in DSS-induced colitis.

### 3.3 FimH promotes effector T cell differentiation

**Figure 3:**
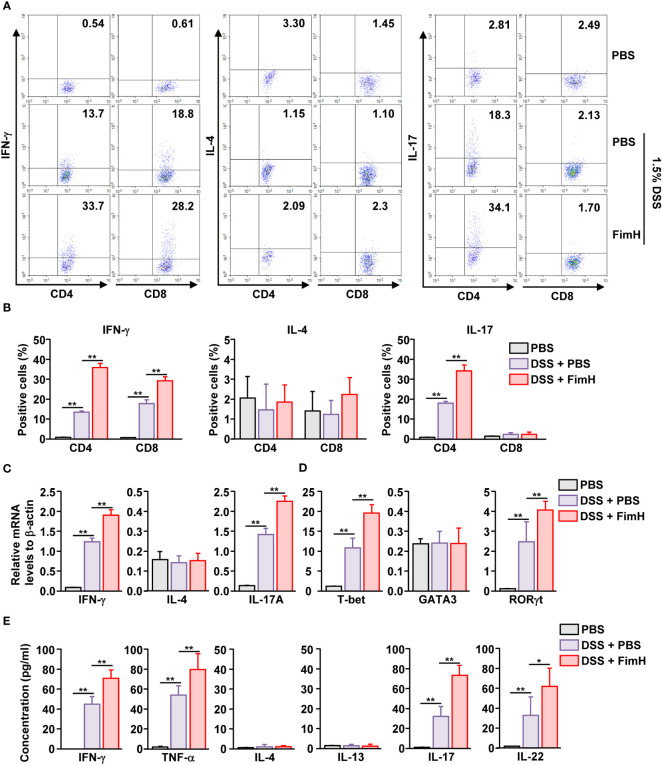
FimH- induced Th1 and Th17 immune response activation in the colon. C57BL/6 mice were administered PBS, 1.5% DSS water, and FimH + DSS water for five days. **(A)** Intracellular productions of IFN-γ, IL-4, and IL-17 were analyzed using flow cytometry. **(B)** Mean positive of IFN-γ-, IL-4-, and IL-17-producing cells. **(C, D)** Indicated mRNA levels were analyzed using real-time PCR. **(E)** Colon single cells were incubated for 24 h, and the concentration of indicated cytokines was quantified using an enzyme-linked immunosorbent assay (ELISA). Data are the average or representative of the analysis of six independent samples (n = 6), *p < 0.05, **p < 0.01.

DSS-induced colitis develops with the involvement of Th1 and Th17 cells (17, 18, 24). Since FimH promoted the exacerbation of DSS-induced colitis, we examined whether FimH elicited differentiation of Th1 and Th17 cells in DSS-induced colitis. The colon was harvested five days after DSS administration, and colon CD4 and CD8 T cells were defined as shown in **Figure S2A**. IFN-γ-producing CD4 and CD8 T cells were considerably increased in the colon of DSS-administrated mice, further elevated by FimH treatment (**Figures 3A, B**). In addition, IL-17-producing CD4 T cells were dramatically increased by FimH administration compared to those in DSS water mice (**Figures 3A, B**). Consistent with intracellular cytokine production, the mRNA levels of IFN-γ and IL-17 were substantially upregulated by FimH (**Figure 3C**). Furthermore, the signature transcription factors of Th1 and Th17, T-bet, and RORγt levels were consistently increased in DSS water mice by FimH compared to that of the PBS control (**Figure 3D**). In the colon single-cell culture condition, the levels of Th1 cells producing IFN-γ and TNF-α in DSS-colitis were considerably increased by FimH administration (**Figure 3E**). Th17 cells produced IL-17 and IL-22 in the culture medium of colon cells, which were considerably upregulated by FimH compared to those of DSS-induced colitis (**Figure 3E**). Thus, these data suggest that FimH upregulates Th1 and Th17 cell activation.

### 3.4 FimH does not directly contribute to Th1 and Th17 differentiation

**Figure 4:**
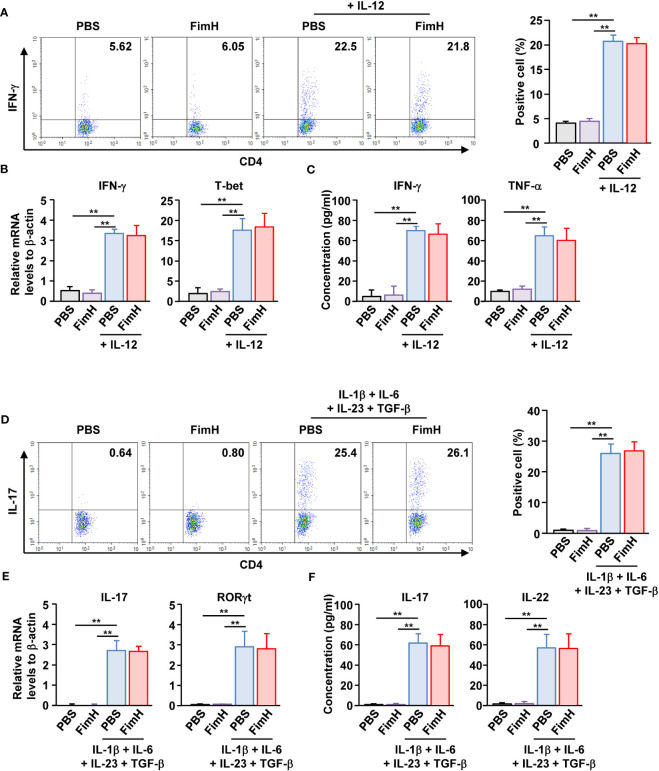
FimH did not directly promote Th1 and Th17 differentiation. **(A–C)** Isolated naïve CD4 T cells were stimulated with anti-CD3/28 antibodies in the presence or absence of IL-12. The cells were further treated with PBS or 20 μg/mL FimH and incubated for three days. **(A)** Intracellular IFN-γ production was analyzed using flow cytometry (left panel). Mean percentage of IFN- γ-positive cells (right panel). **(B)** mRNA levels of IFN-γ and T-bet. **(C)** Concentration of IFN-γ and TNF-α in a cultured medium. **(D–F)** Naïve CD4 T cells were differentiated into Th17 cells by anti-CD3/CD28 antibodies and indicated cytokines. The cells were treated with PBS or 20 μg/mL FimH. **(D)** Intracellular IL-17 production levels (left panel). Mean percentage of IL-17-producing cells (right panel). **(E)** mRNA levels of IL-17 and RORγt. **(F)** Concentration of IL-17 and IL-22 in the cultured medium. Data are the average or representative of the analysis of six independent samples (n = 6), **p < 0.01.

Since FimH administration enhanced Th1 and Th17 immune responses in DSS-induced colitis mice, we further examined whether FimH can directly induce the activation and differentiation of these T cells. Isolated naïve CD4 T cells were differentiated into Th1 cells by stimulation with an anti-CD3/28 antibody and IL-12. Th1 -differentiated cells produced increased levels of IFN-γ. In contrast, FimH treatment did not induce additional intracellular production of IFN-γ in CD4 T cells (**Figure 4A**). FimH did not enhance the mRNA levels of IFN-γ and T-bet compared to those in PBS-treated Th1 cells (**Figure 4B**). Furthermore, the concentrations of IFN-γ and TNF-α in the culture medium of FimH-treated Th1 cells did not increase compared with those produced by PBS-treated Th1 cells (**Figure 4C**). Consistent with the effects of FimH on Th1 cells, FimH treatment did not upregulate intracellular IL-17 production in Th17 cells (**Figure 4D**). The mRNA and cytokine production were not upregulated in Th17 cells by FimH compared to that in PBS-treated Th17 cells (**Figures 4E, F**). Thus, these data indicate that FimH does not directly induce T cell differentiation and activation.

### 3.5 FimH promotes CD11b^+^CD103^-^ DC activation

**Figure 5:**
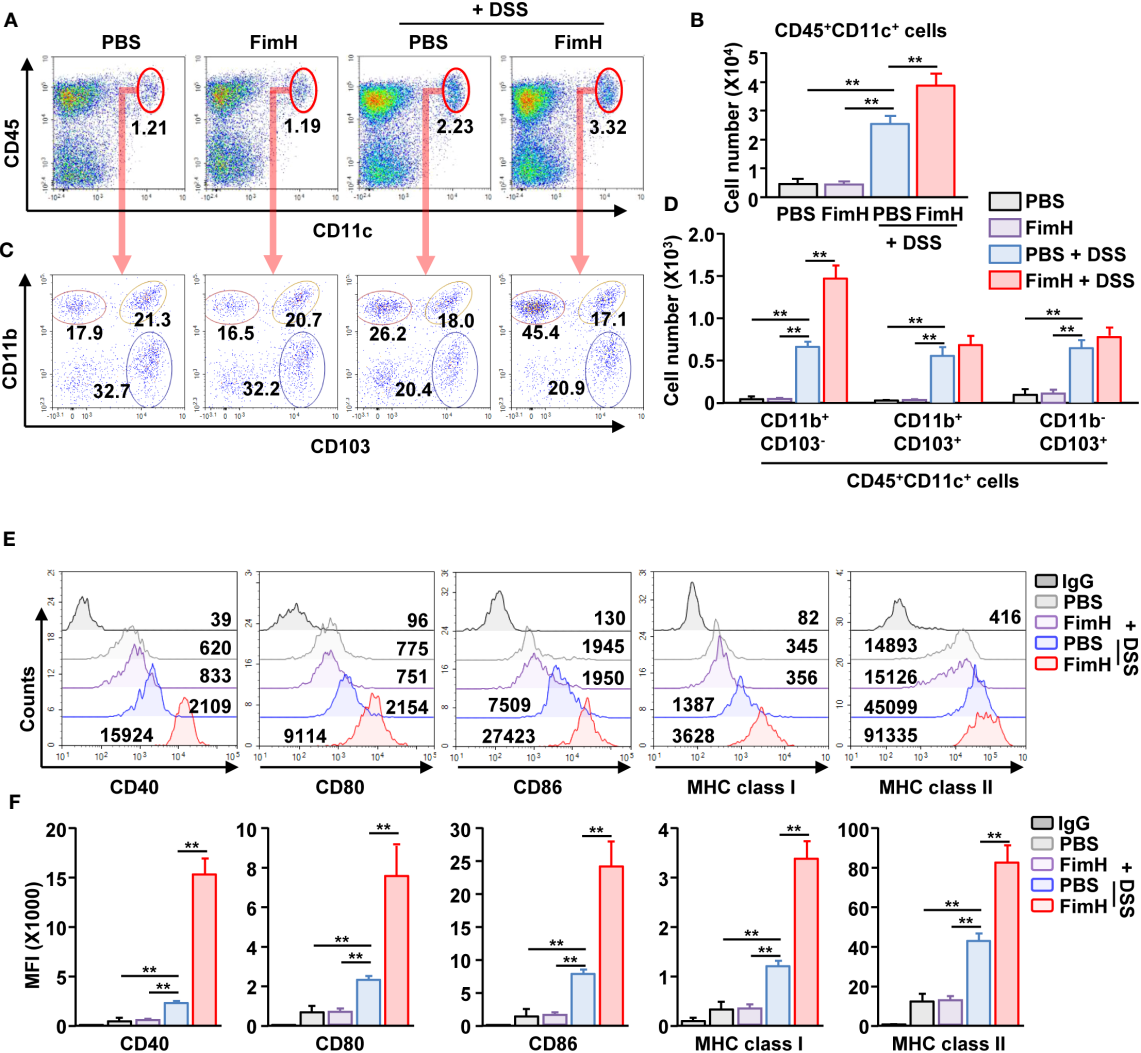
FimH- induced CD11b^+^CD103^-^ DC activation in the mesenteric lymph node (mLN). C57BL/6 mice were orally administered PBS or 10 mg/kg FimH with drinking water containing 1.5% DSS. After three days of drinking DSS water, mLNs were harvested. **(A)** Frequency and population of CD11c^+^CD45^+^ cells in mLN. **(B)** Absolute number of CD11c^+^CD45^+^ cells in mLN. **(C)** CD11c^+^CD45^+^ cells are further divided into CD11b^+^CD103-, CD11b^+^CD103^+^, and CD11b^-^CD103^+^ cells. **(D)** Total number of CD11b^+^CD103^-^, CD11b^+^CD103^+^, and CD11b^-^CD103^+^ cells in CD11c^+^CD45^+^ mLN cells. **(E)** Surface activation marker expression in CD11b^+^CD103^-^ DCs was analyzed using flow cytometry. **(F)** The mean fluorescence intensity (MFI) of the indicated surface markers was shown. Data are the average or representative of the analysis of six independent samples (n = 6), **p < 0.01.

To identify the target cells of FimH for colitis exacerbation, we analyzed the DC activation by FimH. Because the DC population was minor in the colon, mLN DCs were analyzed. We observed that DSS administration increased the number and frequency of CD11c^+^ DCs, further increased by FimH treatment in DSS-induced colitis (**Figures 5A, B**). The number of all types of DC subtypes in the intestine was increased in DSS-induced colitis; however, only CD11b^+^CD103^-^ DCs were additionally increased by the FimH administration (**Figures 5C, D**). Next, the activity marker expression in the DCs was analyzed. The FimH treatment in CD11b^+^CD103^-^ DCs remarkably increased DSS-induced upregulation of co-stimulatory and MHC molecules (**Figures 5E, F**). In addition, FimH treatment induced phosphorylation of ERK, JNK, and p38 in CD11b^+^CD103^-^ DCs, indicating DC maturation signaling pathway activation (**Figure S3**). However, the expression of these activation markers did not increase in CD11b^+^CD103^+^ and CD11b^-^CD103^+^ DCs after DSS and FimH treatment (**Figure S4**). These data indicate that FimH induces CD11b^+^CD103^−^ DC activation.

### 3.6 FimH-activated CD11b^+^CD103^-^ DCs produced pro-inflammatory cytokines

**Figure 6:**
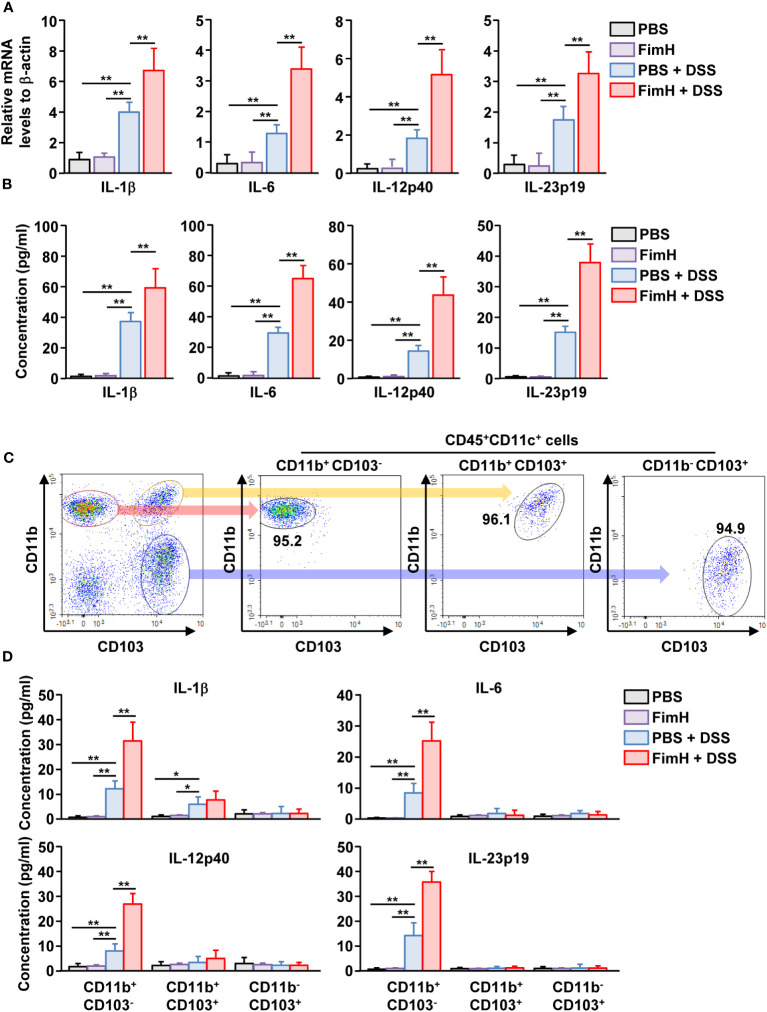
FimH directly stimulated CD11b^+^CD103^-^DCs. **(A)** Indicated mRNA levels in mLN cells were analyzed using real-time PCR on day 3 of DSS and FimH treatment. **(B)** Single cells of mLN were cultured for 24 h. The concentration of indicated cytokines in the cultured medium was quantified using an enzyme-linked immunosorbent assay (ELISA). **(C, D)** The mice were euthanized three days after DSS and FimH treatment, and mLNs were harvested. **(C)** Purified CD11b^+^CD103^-^, CD11b^+^CD103^+^, and CD11b^-^CD103^+^ cells in CD11c^+^CD45^+^ mLN cells. **(D)** CD11b^+^CD103^-^, CD11b^+^CD103^+^, and CD11b^-^CD103^+^ cells were cultured for 24 h, and the concentrations of IL-1β, IL-6, IL-12p40, and IL-23p19 were measured in the cultured medium. Data are the average or representative of the analysis of six independent samples (n = 6), *p < 0.05; **p < 0.01.

To determine the function of DC subsets, cytokine production was examined in mLN cells of mice treated with DSS and FimH. The mLN cells in DSS colitis showed increased mRNA and protein levels of IL-1β, IL-6, IL-12, and IL-23, which were considerably increased by additional treatment with FimH (**Figures 6A, B**). To confirm cytokine production according to mLN DC subsets, we sorted and cultured the subsets of DCs from the mLN of mice treated with DSS and FimH (**Figure 6C**). The IL-1β production levels in the culture medium were considerably increased in CD11b^+^CD103^-^ and CD11b^+^CD103^+^ DCs (**Figure 6D**). The IL-1β production levels were remarkably increased in the culture medium of CD11b^+^CD103^-^ DCs by FimH, whereas FimH treatment did not enhance these levels in CD11b^+^CD103^+^ DCs in the mLN of DSS-induced colitis (**Figure 6D**). In addition, only CD11b^+^CD103^-^ DCs secreted IL-6, IL-12p40, and IL-23p19 from the mLN of DSS-colitis mice, which was further enhanced by the treatment with FimH (**Figure 6D**). Thus, these data suggest that FimH stimulates pro-inflammatory cytokine production in CD11b^+^CD103^-^ DCs in DSS-induced colitis.

### 3.7 FimH-activated CD11b^+^CD103^-^ DCs induce Th1 and Th17 cell differentiation

**Figure 7:**
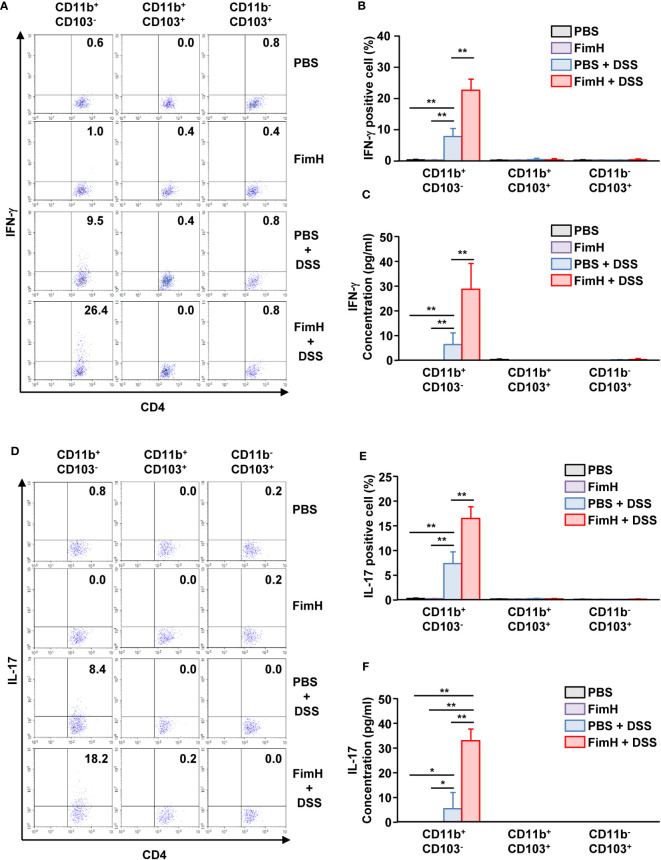
FimH and DSS stimulated CD11b^+^CD103^-^ DCs and elicited Th1 and Th17 immune responses. C57BL/6 mice were orally administered PBS or 10 mg/kg FimH with drinking water containing 1.5% DSS for three days. CD11b^+^CD103^-^, CD11b^+^CD103^+^, and CD11b^-^CD103^+^ DCs were isolated and co-cultured with naïve CD4 T cells for three days. **(A)** Intracellular IFN-γ production in CD4 T cells. **(B)** Mean percentage of IFN-γ- positive cells in CD4 T cells. **(C)** Concentration of IFN-γ in the culture medium. **(D)** IL-17-producing CD4 T cells were analyzed using flow cytometry. **(E)** Mean percentage of IL-17-producing cells in CD4 T cells. **(F)** Concentration of IL-17 in the culture medium was quantified using an enzyme-linked immunosorbent assay (ELISA). Data are the average or representative of the analysis of six independent samples (n = 6), *p < 0.05, **p < 0.01.

To confirm the T cell differentiation function of CD11b^+^CD103^-^ DCs stimulated by FimH, CD11b^+^CD103^-^ DCs were isolated from the mLN of FimH-treated colitis mice and co-cultured with naïve T cells. CD11b^+^CD103^-^ DCs isolated from the mLN of colitis mice induced IFN-γ production in T cells. The expression of IFN-γ was further enhanced in mLN CD11b^+^CD103^-^ DCs from mice treated with FimH and DSS compared to that produced by PBS-treated CD11b^+^CD103^-^ DCs (**Figures 7A, B**). Consistent with the intracellular production levels of IFN-γ in CD4 T cells, the concentration of IFN-γ in the culture medium of CD4- and FimH-stimulated CD11b^+^CD103^-^ DCs was considerably upregulated compared to that in PBS-treated CD11b^+^CD103^-^ DCs from DSS-colitis mice (**Figure 7C**). Next, we examined the effect of FimH-stimulated mLN DC subsets on Th17 cell activation and identified that CD11b^+^CD103^-^ DCs from FimH- and DSS-treated mice mLN substantially increased the intracellular production of IL-17 in CD4 T cells compared to that of PBS- and DSS-treated CD11b^+^CD103^-^ DCs (**Figures 7D, E**). IL-17 production levels in the co-culture medium of FimH and DSS-stimulated CD11b^+^CD103^-^ DCs and naïve T cells were remarkably elevated compared to those induced by co-culture of DSS-stimulated CD11b^+^CD103^-^ DCs and naïve T cells (**Figure 7F**). These results demonstrated that FimH-stimulated CD11b^+^CD103^-^ DCs in the mLN and DSS directly induced Th1 and Th17 immune responses.

## 4 Discussion

FimH may primarily cause AIEC-induced colitis (38, 39). In addition, several studies have reported that FimH induces immune activity (12–14, 40). Therefore, blocking FimH could be a strategy to prevent AIEC-induced colitis (35, 39). However, direct FimH-induced colitis has not yet been reported. Accordingly, in this study, oral or *i.p.* administration of recombinant FimH did not induce colitis. However, colitis in mice that consumed 1.5% DSS water was exacerbated by oral FimH treatment. This may be due to FimH infiltration into the epithelium of the large intestine damaged by DSS, which induces strong immune activity. Therefore, rather than directly inducing colitis, FimH may exacerbate colitis when the colonic epithelial cells are damaged.

Colitis is caused by food poisoning, bacteria, viruses, parasites, and genetic effects that promote damage to the epithelial cells of the colon (15). The potential of AIEC to directly induce colitis by invading colonic epithelial cells has been well documented (41). However, studies regarding the causes of the aggravation of relatively mild colitis are lacking. In this study, we suggest that FimH, an adhesion protein of *E. coli*, exacerbates colitis caused by damage to the colon wall. In most colitis studies using DSS, 3–5% DSS is used to induce colitis in mice (42, 43). However, colitis induced in this way is an extreme situation, and in a few cases, mice die after 7 days (36, 42, 43). We used 1.5% DSS to induce relatively mild inflammation and additionally administered FimH to study the function of FimH and observed that FimH could exacerbate colitis. Therefore, colitis caused by AIEC may have been aggravated by the inflammation caused by FimH in AIEC after damage to the colonic epithelium by other factors.

The large intestine can be exposed to various external factors and is regulated by the immune response against these factors (44). However, colitis can be induced by several factors. Colitis is a disease in which activated immune cells attack the epithelial cells of the colon to destroy their function, and the disease progresses with the involvement of these immune cells (45). Among these immune cells, activated CD4 T, Th1, and Th17 cells were observed in the epithelial layer of the colon of patients with colitis (18, 24). FimH was fed to mice drinking DSS water, and it was shown that the immune response of Th1 and Th17 cells in the large intestine increased. Although Th1 and Th17 are competitively induced in general immunity, Th1 can induce a Th17 immune response in the colon (46, 47). In addition, we observed that FimH induced T-cell activity by directly stimulating colonic DCs. DCs expressing CD103 induce immune tolerance in the colon, which induces the differentiation of regulatory T cells (23). In contrast, CD11b-expressing DCs are inflammatory DCs that induce T-cell activation (48). In line with this, in mice treated with FimH, CD11b^+^CD103^-^ DCs in the mLN were activated, and these cells induced T cell activation. However, we could not determine why other DC subtypes in the mLN were inactive. This may be due to the expression of Toll-like receptor 4 (TLR4), which stimulates TLR4 in mice and humans (12). Therefore, it is necessary to conduct additional experiments to study TLR expression according to the DC subtypes.

Immune stimulators are commonly used to improve the effectiveness of vaccines (3, 21). When antigenic proteins lack immunogenicity, specific immune activity against the antigen may be induced by administering them in combination with an immune stimulator (4). In previous studies, we suggested that FimH acts as an immune stimulator for enhancing cancer vaccines (12). In addition, the results posited that the anticancer effect of the immune checkpoint antibody can be improved by FimH (12). However, the results of this study suggest an important side effect of FimH in the body. FimH has sufficient potential for use as a protein-based immune stimulator, and its use in patients with inflammatory diseases should be considered. However, it does not directly induce inflammatory diseases like other immune enhancers.

## 5 Conclusions

The use of immune stimulators may be accompanied by side effects, such as inflammatory disease induction. FimH, an immune stimulator discovered in previous studies, did not induce inflammation of the colon by intraperitoneal or oral administration; however, it aggravated the degree of inflammation in colitis-induced mice. FimH aggravated colitis by inducing Th1 and Th17 immune responses, mediated by CD11b^+^CD103^-^ DC activation in the colon. Therefore, this study demonstrated the side effects of FimH and indicated that the use of FimH in patients with colitis can aggravate colitis.

## Data availability statement

The raw data supporting the conclusions of this article will be made available by the authors, without undue reservation.

## Ethics statement

The animal experiments were approved by the Ethics Committee of Shanghai Public Health Clinical Center (mouse protocol number: 2021-A070-01) and the Asan Medical Center (authorization no. 2023-04-041). The study was conducted in accordance with the local legislation and institutional requirements.

## Author contributions

WZ: Data curation, Writing – original draft, Formal analysis, Investigation. EA: Data curation, Formal analysis, Investigation, Methodology, Writing – review & editing. SK: Data curation, Formal analysis, Investigation, Methodology, Writing – review & editing. HP: Formal analysis, Investigation, Methodology, Writing – review & editing. PL: Conceptualization, Data curation, Project administration, Resources, Supervision, Writing – review & editing. JJ: Conceptualization, Data curation, Supervision, Writing – review & editing, Writing – original draft.
