# Supplementary material for: Escherichia coli adhesion protein FimH exacerbates colitis via CD11b+CD103- dendritic cell activation
Source: Front Immunol. 2023 Nov 22;14:1284770. doi: 10.3389/fimmu.2023.1284770 (PMC10703180; doi:10.3389/fimmu.2023.1284770)
Supplement: Supplementary file 1 [file DataSheet_1.docx]

*Supporting information*

**Escherichia coli adhesion protein FimH exacerbated colitis by activation of CD11b^+^CD103^-^ dendritic cells**

Wei Zhang^1†^, Eun-Koung An^2†^, So-Jung Kim^2†^, Hae-Bin Park^2^, Peter C.W. Lee^3*^ and Jun-O Jin^2*^

| IFN-γ | forward | 5′-GGATGCATTCATGAGTATTGC-3′ |
| --- | --- | --- |
|  | reverse | 5′-CTTTTCCGCTTCCTGAGG-3′ |
| IL-4 | forward | 5′-ACAGGAGAAGGGACGCCAT-3′ |
|  | reverse | 5′-GAAGCCCTACAGACGAGCTCA-3′ |
| IL-17A | forward | 5′-GCGCAAAAGTGAGCTCCAGA-3′ |
|  | reverse | 5′-ACAGAGGGATATCTATCAGGG-3′ |
| T-bet | forward | 5′-CAACAACCCCTTTGCCAAAG-3′ |
|  | reverse | 5′-TCCCCCAAGCATTGACAGT-3′ |
| GATA3 | forward | 5′-AGAACCGGCCCCTTATCAA-3′ |
|  | reverse | 5′-AGTTCGCGCAGGATGTCC-3′ |
| RORγt | forward | 5′-CCGCTGAGAGGGCTTCAC-3′ |
|  | reverse | 5′-TGCAGGAGTAGGCCACATTACA-3′ |
| IL-1β | forward | 5′-TGTAATGAAAGACGGCACACC-3′ |
|  | reverse | 5′-TCTTCTTTGGGTATTGCTTGG-3′ |
| IL-6 | forward | 5′-ACGATGATGCACTTGCAGA-3′ |
|  | reverse | 5′-GAGCATTGGAAATTGGGGTA-3′ |
| IL-12p40 | forward | 5′-CACATCTGCTGCTCCACAAG-3′ |
|  | reverse | 5′-CCGTCCGGAGTAATTTGGTG-3′ |
| IL-23p19 | forward | 5′-CTCTCGGAATCTCTGCATGC-3′ |
|  | reverse | 5′-ACCATCTTCACACTGGATACG-3′ |

**Table S1 Primer sequences for real-time PCR**

^
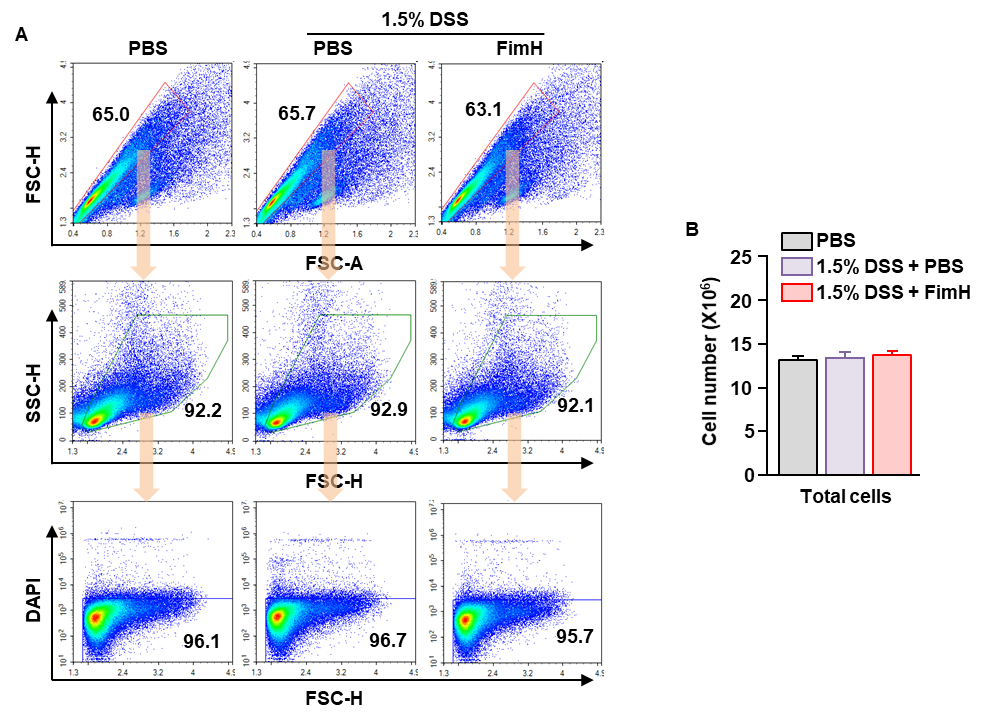
^

**Figure S1. Analysis of colon infiltrated cells using flow cytometry.** Colon single-cell suspension was prepared, as shown in the Methods section. **(A)** Live cell gating strategy. **(B)** Total cell number in the colon.


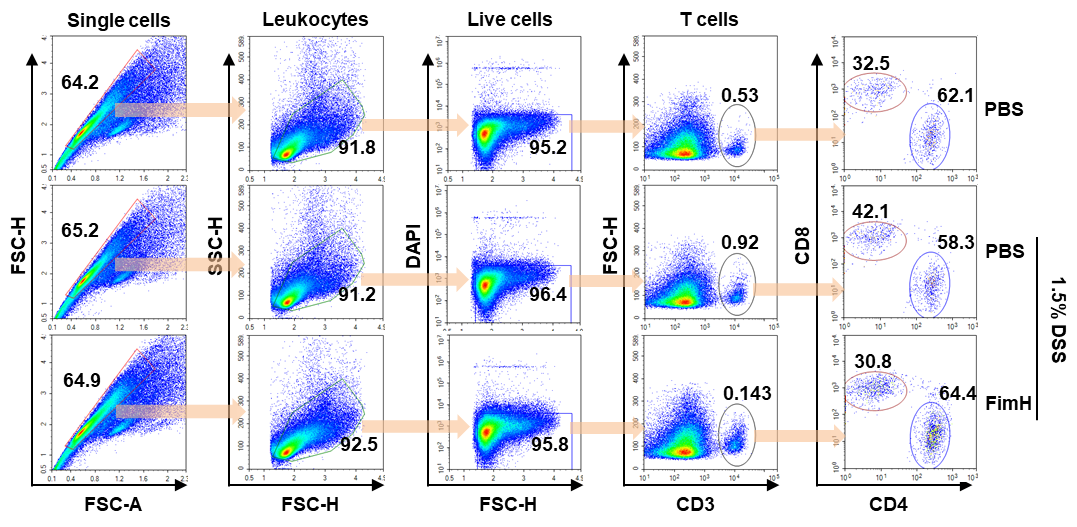


**Figure S2. Flow cytometry analysis of colon infiltrated T cells.** C57BL/6 mice were administered PBS, 1.5% DSS water, and FimH + DSS water for five days. CD4 and CD8 T cells were gated from live leukocytes in the colon single-cell suspension.


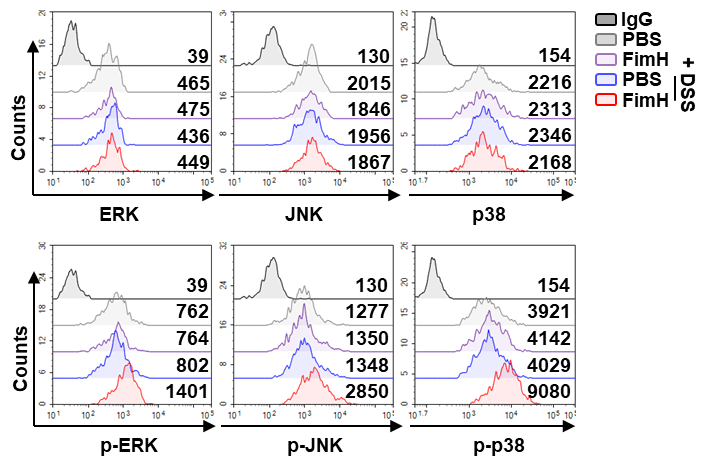


**Figure S3. Phosphorylation of MAPK in the CD11b^+^CD103^-^ DCs by FimH.** C57BL/6 mice were orally administered PBS or 10 mg/kg FimH with drinking water containing 1.5% DSS. After three days of drinking DSS water, mLNs were harvested. Indicated intracellular protein levels were analyzed using flow cytometry in CD11b+CD103- DCs.


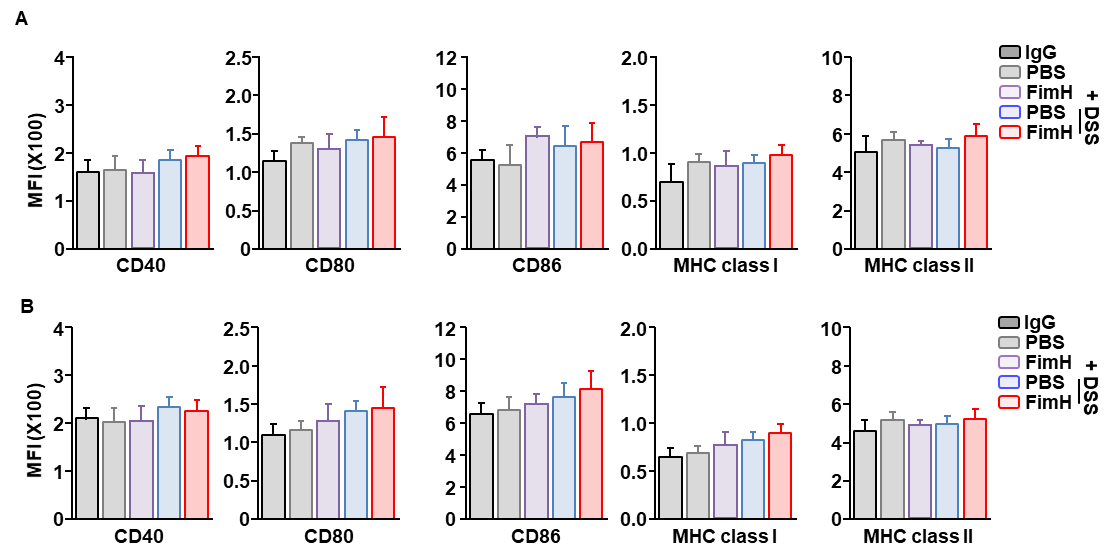


**Figure S4.** **FimH did not activate CD11b^+^CD103^+^ nor CD11b^-^CD103^+^ DCs in the mesenteric lymph node (mLN).** The mLN cells were harvested as indicated in Figure 5. **(A, B)** Surface activation markers in **(A)** CD11b^+^CD103^+^ and **(B)** CD11b^+^CD103^+^ DCs were analyzed using flow cytometry.

.
